# Supplementary material for: Association between Variants of the TRPV1 Gene and Body Composition in Sub-Saharan Africans
Source: Genes (Basel). 2024 Jun 7;15(6):752. doi: 10.3390/genes15060752 (PMC11202968; doi:10.3390/genes15060752)
Supplement: Supplementary file 1 [file genes-15-00752-s001.zip › Figure S1.pdf]

Dietary habits

Personal info

ID: ________________________ Sex_______

Place of birth: _______________________ Date of birth ____________

Age: __________ Weight: ___________ Height: __________

Parents place of birth: Father ____________________ Mother____________________

Smoker: ☐YES ☐ NO

Respiratory problems: ☐YES☐ NO Which ones: ____________________________________

Diseases (Cardiovascular, Diabetes, Obesity...)☐YES☐ NO Which ones:___________________________

Food intolerances: ☐YES☐ NO Which ones: ____

Do you practise physical activity? : ☐YES☐ NOIf you do, competitively? ☐YES☐ NO

How often do you eat this foods?

Chilly pepper

1 -never☐

2–At least 2-3 times a month☐

3–At least 2-3 times a week☐

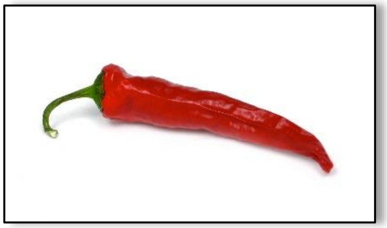

Pepper

1–never☐

2 - At least 2-3 times a month ☐

3 - At least 2-3 times a week ☐

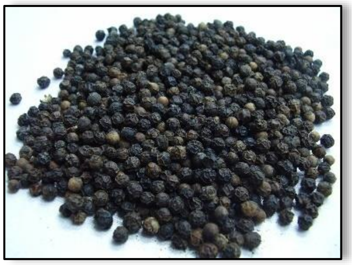

Garlic

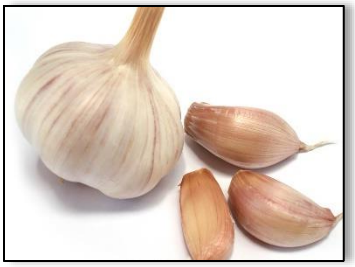

1 - never☐

2 - At least 2-3 times a month ☐

3 - At least 2-3 times a week ☐

4 - Cucked☐ Raw ☐

Onion

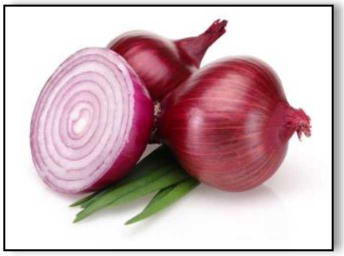

1 - never☐

2 - At least 2-3 times a month ☐

3 - At least 2-3 times a week ☐

4- Cucked☐ Raw ☐

Ginger

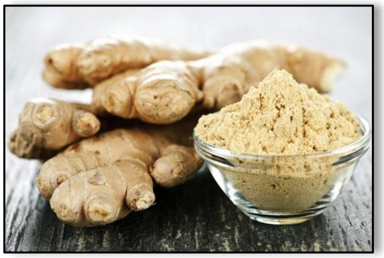

1–never☐

2 - At least 2-3 times a month ☐

3 - At least 2-3 times a week ☐

Turmeric

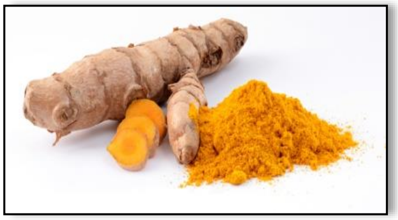

1 - never☐

2 - At least 2-3 times a month ☐

3 - At least 2-3 times a week ☐

Paradise grains/Guinea pepper/Maniguette

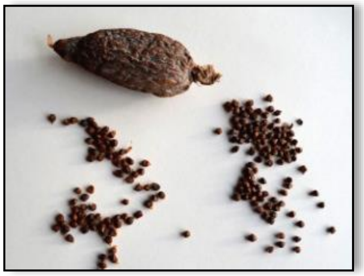

1 - never☐

2 - At least 2-3 times a month ☐

3 - At least 2-3 times a week ☐

Alcoholic drinks

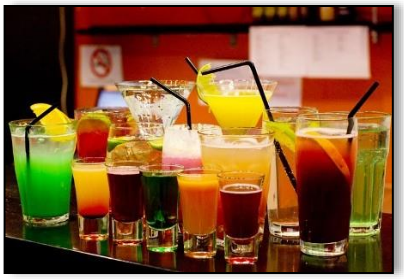

1 - never☐

2 - At least 2-3 times a month ☐

3 - At least 2-3 times a week ☐

Mint

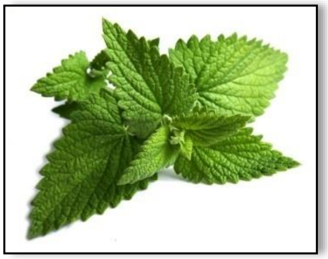

1 - never☐

2 - At least 2-3 times a month ☐

3 - At least 2-3 times a week ☐

Chewing gum/breath mints

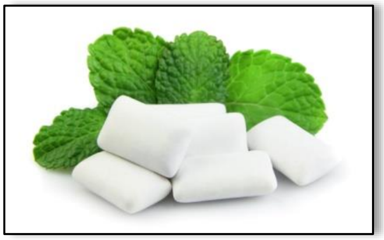

1 - never☐

2 - At least 2-3 times a month ☐

3 - At least 2-3 times a week ☐

Indicate the tolerance threshold to:

Hot	low ☐	intermediate ☐	high ☐
Cold	low ☐	intermediate ☐	high ☐
Pain	low ☐	intermediate ☐	high ☐

For non-Italian volunteers

Is the diet you follow in Italy different from that you followed before you came live here?In which way?

....

How long have you lived away from your country of origin?

....

In the last ____ years, the use of hot spicy foods:

decreased☐ remained unchanged ☐ increased ☐

In the last ____ years, the use of fresh foods:

decreased ☐ stayed unchanged ☐ increased ☐
