# Supplementary material for: Association between Variants of the TRPV1 Gene and Body Composition in Sub-Saharan Africans
Source: Genes (Basel). 2024 Jun 7;15(6):752. doi: 10.3390/genes15060752 (PMC11202968; doi:10.3390/genes15060752)
Supplement: Supplementary file 1 [file genes-15-00752-s001.zip › Supplementary material_ Figures' captions.pdf]

Supplementary material. Figures' captions

Figure S1. The questionnaire administered to reconstruct dietary habits.

Figure S2. Capsaicin perception thresholds for ITA and SSA groups, regardless of sex.

Figure S3. PROP intensity distribution in relation to BMI. ITA_M and ITA_F on the left hand side, SSA_M and SSA_F on the right hand side.

Figure S4. BIVA tolerance ellipses showing vectors for a) ITA_F (blue square) and SSA_F (green triangles) individuals and b) the average of ITA_F (purple triangle) and SSA_F (white circle) groups. A population of Afro-American women is taken as a reference.

Figure S5. BIVA confidence ellipses showing population mean (vector) and 0.95 confidence interval (ellipse perimeter) for East African (yellow) and West African (green) samples.
