# Supplementary figures and images for: Association between Variants of the TRPV1 Gene and Body Composition in Sub-Saharan Africans
Source: Genes (Basel). 2024 Jun 7;15(6):752. doi: 10.3390/genes15060752 (PMC11202968; doi:10.3390/genes15060752)

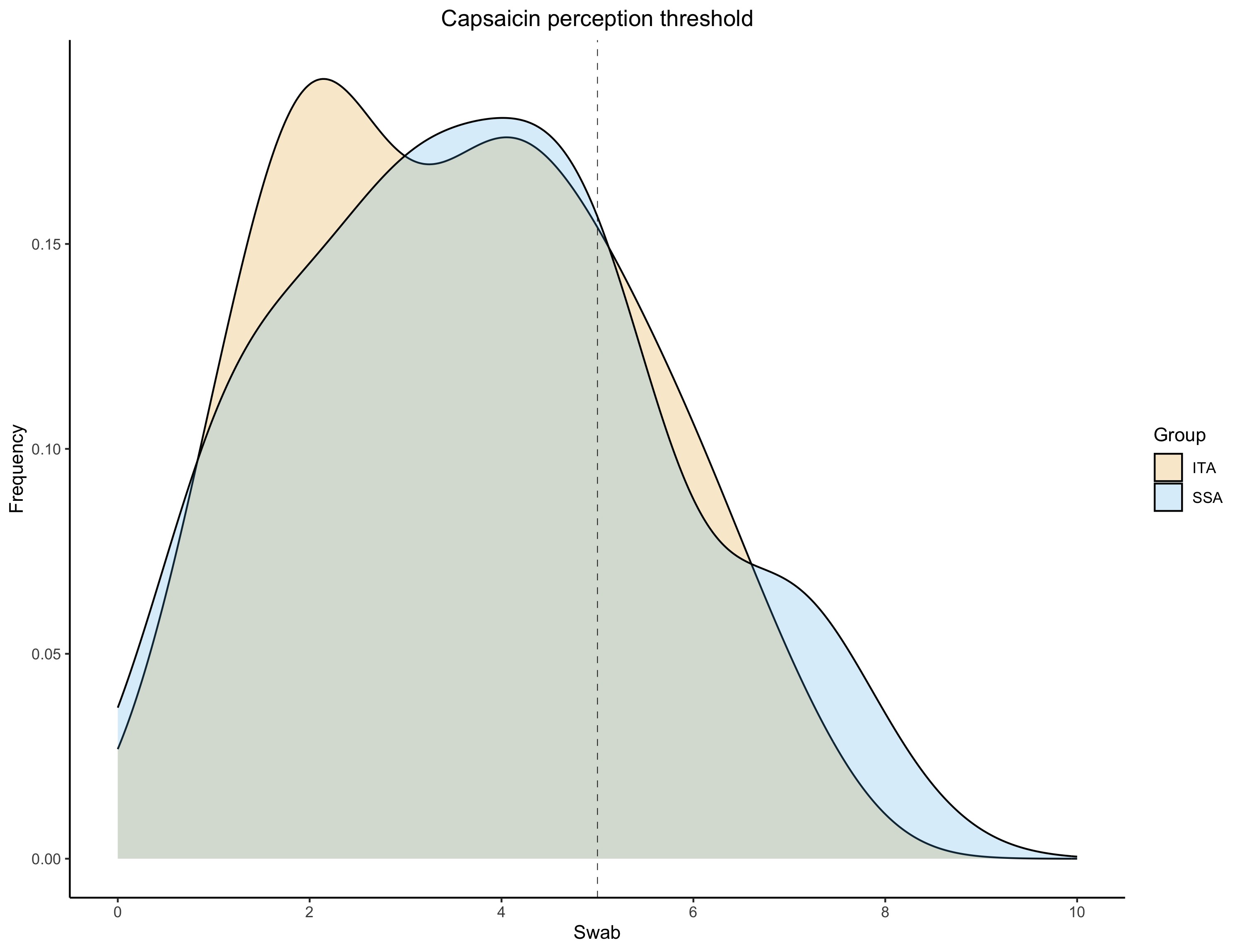

Supplement: Supplementary file 1 [file genes-15-00752-s001.zip › Figure S2.jpg]

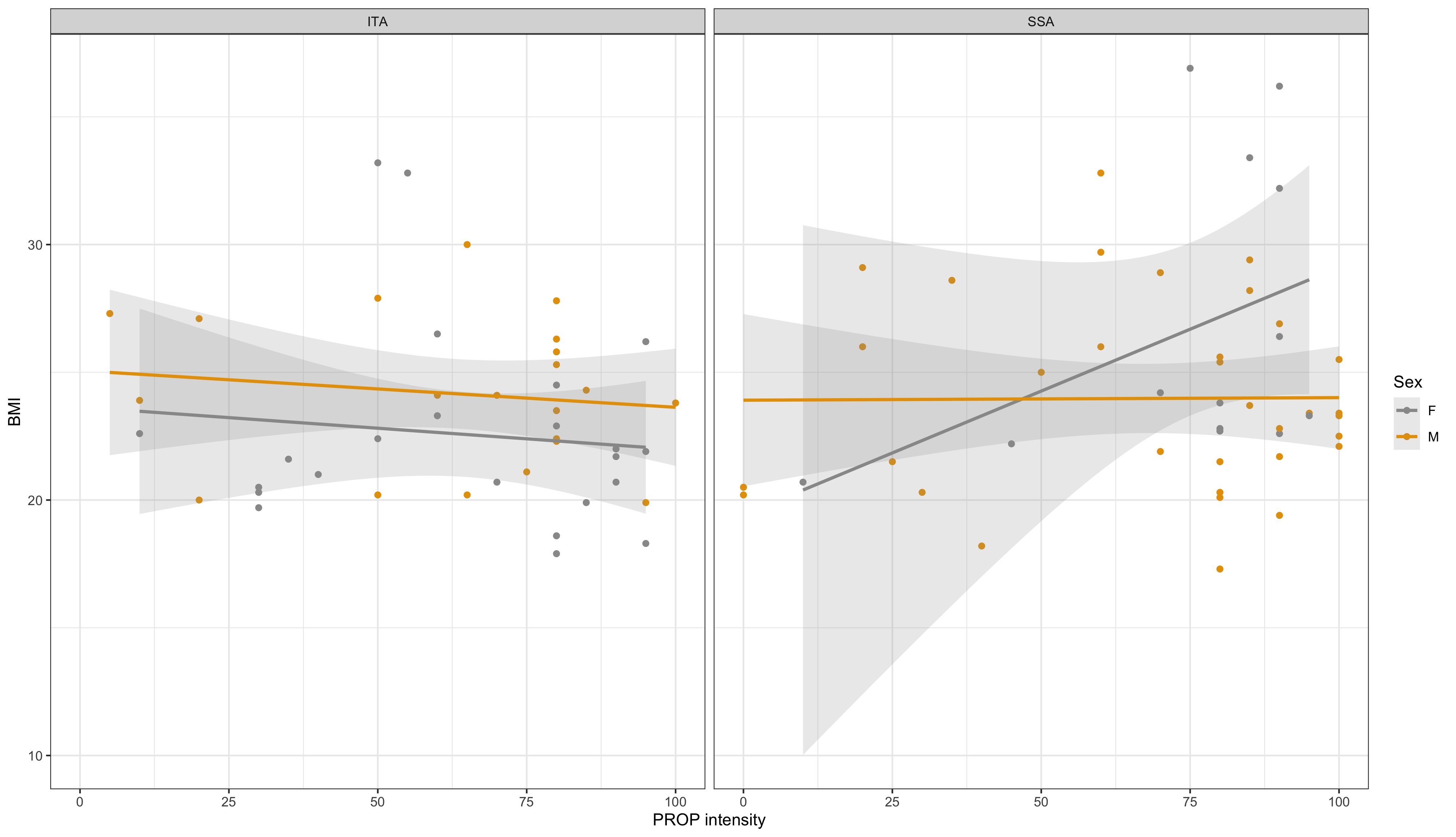

Supplement: Supplementary file 1 [file genes-15-00752-s001.zip › Figure S3.jpg]

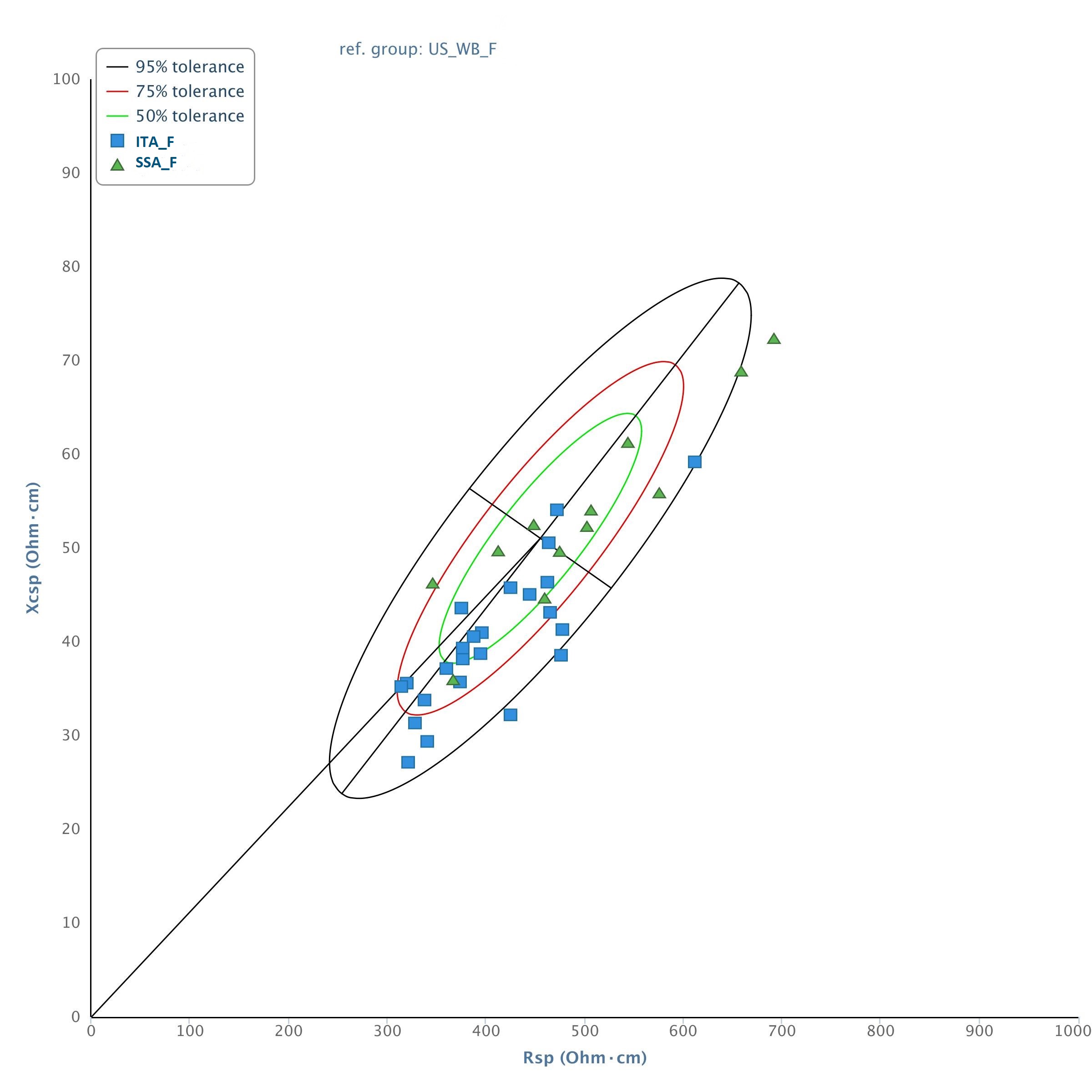

Supplement: Supplementary file 1 [file genes-15-00752-s001.zip › Figure S4a.jpeg]

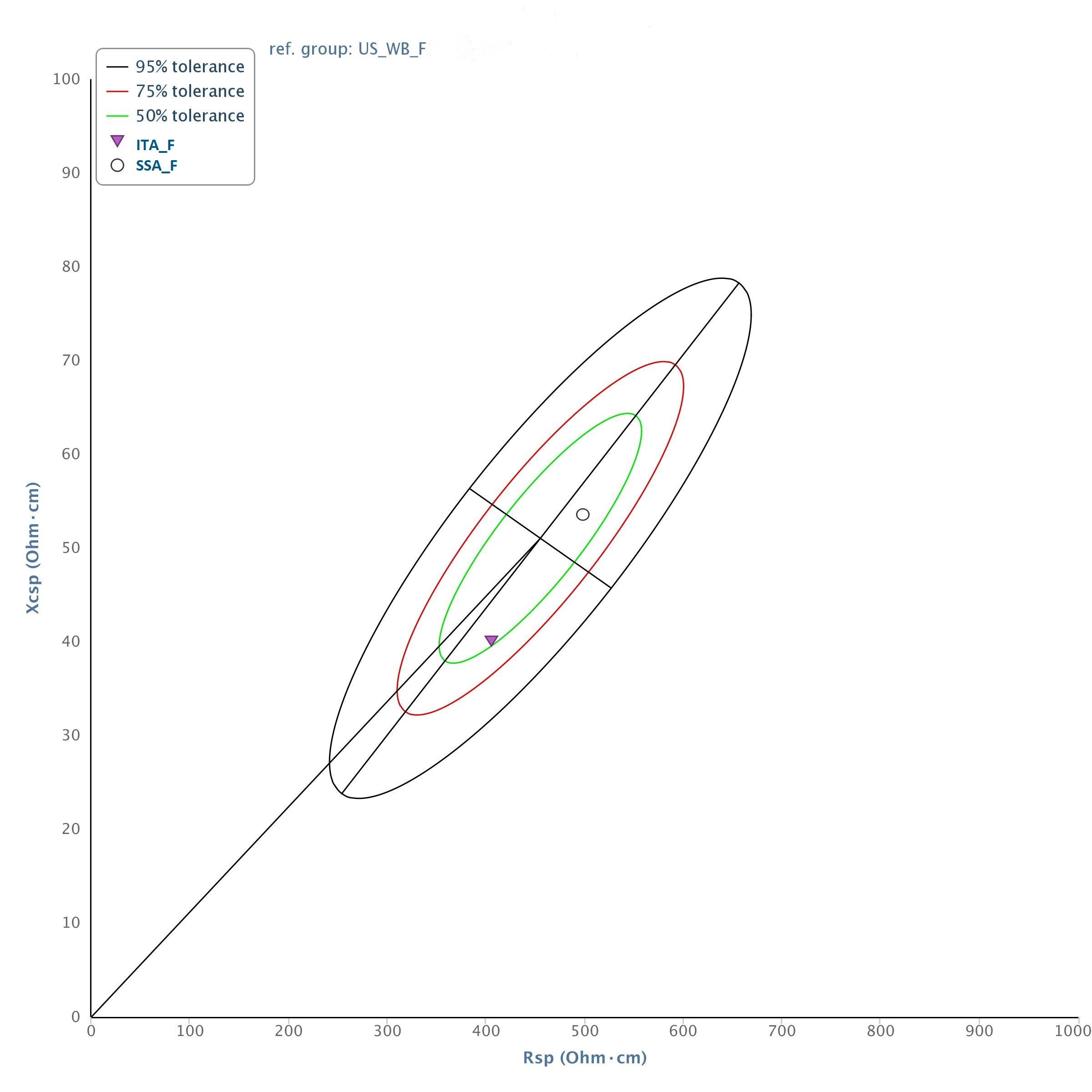

Supplement: Supplementary file 1 [file genes-15-00752-s001.zip › Figure S4b.jpeg]

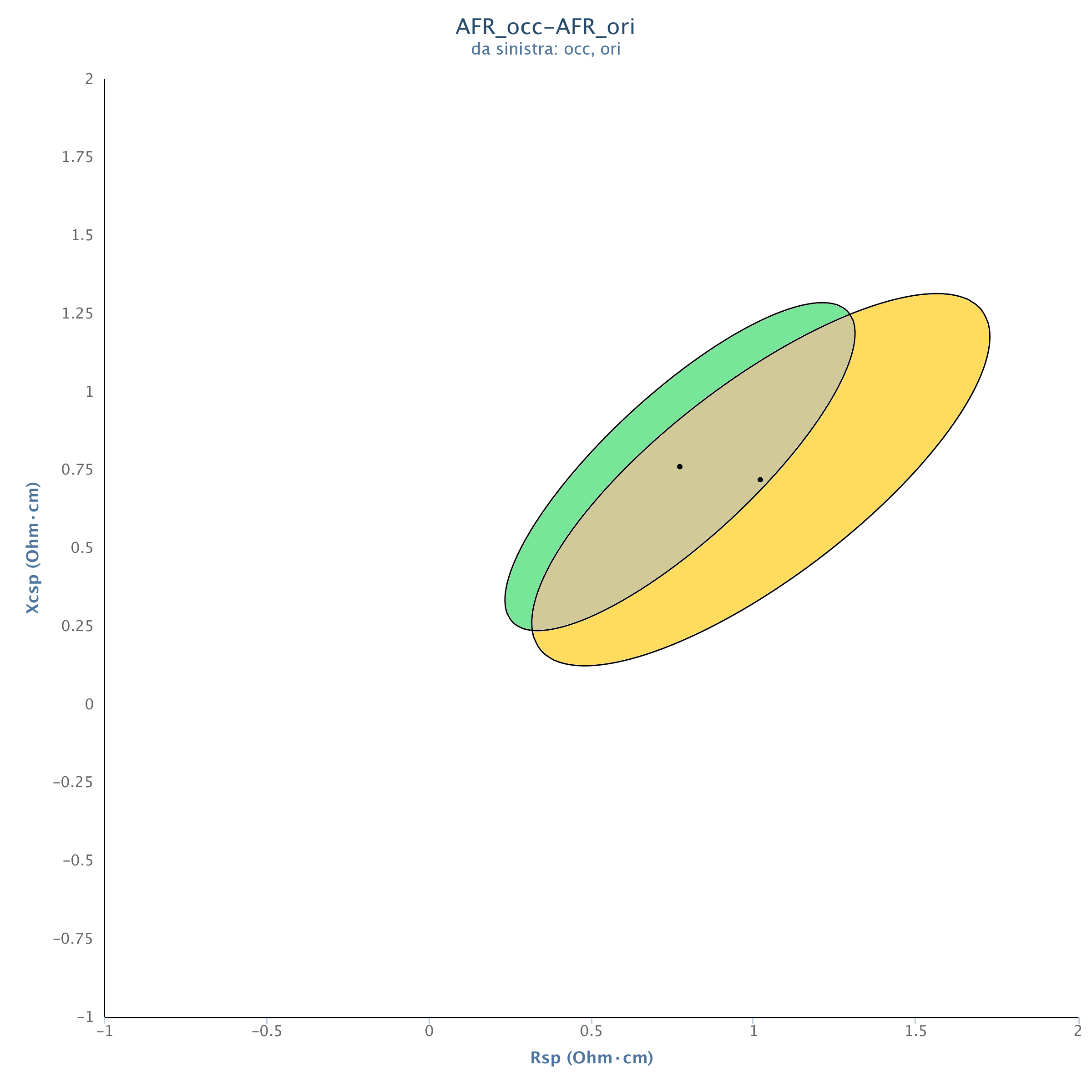

Supplement: Supplementary file 1 [file genes-15-00752-s001.zip › Figure S5.png]
